# Mutations of the Calcium Channel Gene *cacophony* Suppress Seizures in *Drosophila*

**DOI:** 10.1371/journal.pgen.1005784

**Published:** 2016-01-15

**Authors:** Arunesh Saras, Mark A. Tanouye

**Affiliations:** 1 Department of Environmental Science, Policy and Management, Division of Organismal Biology, University of California Berkeley, Berkeley, California, United States of America; 2 Department of Molecular and Cell Biology, University of California Berkeley, Berkeley, California, United States of America; Florey Institute of Neuroscience and Mental Health, AUSTRALIA

## Abstract

Bang sensitive (BS) *Drosophila* mutants display characteristic seizure-like phenotypes resembling, in some aspects, those of human seizure disorders such as epilepsy. The BS mutant *para*^*bss1*^, caused by a gain-of-function mutation of the voltage-gated Na^+^ channel gene, is extremely seizure-sensitive with phenotypes that have proven difficult to ameliorate by anti-epileptic drug feeding or by seizure-suppressor mutation. It has been presented as a model for intractable human epilepsy. Here we show that *cacophony* (*cac*^*TS2*^), a mutation of the *Drosophila* presynaptic Ca^++^ channel α_1_ subunit gene, is a particularly potent seizure-suppressor mutation, reverting seizure-like phenotypes for *para*^*bss1*^ and other BS mutants. Seizure-like phenotypes for *para*^*bss1*^ may be suppressed by as much as 90% in double mutant combinations with *cac*^*TS2*^. Unexpectedly, we find that *para*^*bss1*^ also reciprocally suppresses *cac*^*TS2*^ seizure-like phenotypes. The *cac*^*TS2*^ mutant displays these seizure-like behaviors and spontaneous high-frequency action potential firing transiently after exposure to high temperature. We find that this seizure-like behavior in *cac*^*TS2*^ is ameliorated by 85% in double mutant combinations with *para*^*bss1*^.

## Author Summary

Seizure disorders, such as epilepsy, are a serious health concern because of the large number of patients affected and a limited availability of treatment options. About 10% of the population will have at least one seizure during their lifetime, and 1% will experience persistent, recurrent epileptic seizures. Moreover, for about one-third of patients, epilepsy is intractable with seizures that are not controlled with any available drugs. Genetic seizure suppressors are modifier mutations that are capable of reverting seizure susceptibility to wild type levels when combined with seizure-prone mutants in double mutant individuals. Suppressors are valuable in providing an experimental approach that can provide insight into mechanisms underlying seizure susceptibility. Also, they identify novel gene products that may be targets for therapeutic drug development. In the present study we show that a severe seizure phenotype of the *Drosophila paralytic*^*bss1*^ *(para*^*bss1*^*)* mutant is 90% suppressed by the N-type calcium channel mutation *cacophony*^*TS2*^ *(cac*^*TS2*^*)*. The effect of suppression is not restricted to *para*^*bss1*^, but *cac*^*TS2*^ can also revert seizure-like phenotypes of other *Drosophila* mutants like *easily-shocked (eas)* and *slamdance (sda)*. Thus, *cac*^*TS2*^ acts as a highly potent, general seizure suppressor mutation. A surprising finding in this study is co-suppression: *para*^*bss1*^ also suppresses a seizure phenotype in *cac*^*TS2*^ mutants induced by elevated temperature. A current view of complex diseases such as epilepsy, is that multiple genes and environmental factors can each contribute small, additive effects that can summate to produce a disease state when some threshold value is exceeded. Our findings indicate that different pathogenic ion channel mutations can sometimes form therapeutic combinations with effects that mask one another.

## Introduction

Human seizure disorders are a substantial neurological health problem because of the large number of affected individuals, and heterogeneity underlying the many syndromes. An estimated 1% of the U.S. population, nearly 3 million Americans, is affected by the more than 40 different syndromes that comprise the epilepsies [1,2]. Seizures occur because of an imbalance in excitation and inhibition: excitation can be excessive, inhibition can be inadequate, or both. The resulting seizure activity involves large numbers of neurons firing uncontrollably and synchronously, usually in a rhythmic pattern. Multiple and different molecular aspects of electrical signaling appear to be responsible for the triggering of seizures at the site of initiation or focus, their subsequent spread from the focus to adjacent regions of nervous tissue, and their eventual termination.

In this study, we examine the contribution of basic synaptic transmission to seizure-susceptibility in a *Drosophila* model using mutations of the *cacophony (cac)* gene, responsible for neurotransmitter release. The *cac* gene encodes the α_1_ subunit of the *Drosophila* voltage-gated presynaptic Ca^++^ channel, homologous to the mammalian N-type channel [3–7]. The allele used here, *cac*^*TS2*^, shows conditional and reversible phenotypes dependent on temperature: a behavioral paralysis phenotype and a loss of neurotransmitter release phenotype [5–7]. At restrictive high temperatures, evoked synaptic currents are markedly reduced in *cac*^*TS2*^ mutants, returning to wild-type levels when temperature is lowered to permissive temperatures.

We report here that the *cac*^*TS2*^ mutation affects seizure susceptibility in complex ways including seizure-sensitivity and seizure-resistance, under different conditions. As reported [8], *cac* temperature-sensitive mutants display spontaneous seizure-like activity when shifted to restrictive temperature. We find here that at permissive temperature, *cac*^*TS2*^ is a seizure-resistant mutation and a potent seizure-suppressor. In double mutant combinations with bang-sensitive (BS) seizure-sensitive mutants, *cac*^*TS2*^ is one of the strongest seizure-suppressors that we have identified in the fly, to date. In particular, the *cac*^*TS2*^ mutation is found to ameliorate seizure-like phenotypes in homozygous *para*^*bss1*^, a Na^+^ channel gain-of-function mutation, the most severe of *Drosophila* seizure-sensitive mutations [9,10] and resembling, in some aspects, Na^+^ channel loss-of-function mutations responsible for intractable epilepsy [11,12]. The *cac*^*TS2*^ mutation is a good suppressor of *para*^*bss1*^ phenotypes comparable to *maleless*^*napts*^ and stronger than heat-treated *shibire*^*ts1*^ and *gilgamesh* (Table 1)[13,14,15,16].

**Table 1:** Suppression of behavioral bang-sensitive paralytic phenotypes by *cac*^*TS2*^ and *cacRNAi*. Flies of the appropriate genotype (n ≥ 100) were stimulated by mechanical stimulation delivered by a vortex mixer (10 sec at maximum speed). The number of flies undergoing paralysis was counted to determine the percent paralysis and the percent suppressed (unparalyzed).

Genotype	% BS	% Suppression
*sda* (RT)	100	0
*sda* (HS)	100	0
*eas* (RT)	100	0
*eas* (HS)	100	0
*para*^*bss1*^ (RT)	100	0
*para*^*bss1*^ (HS)	100	0
*para*^*bss1*^*/+* (RT)	62	38
*cac*^*TS2*^*/Y; sda* (RT)	12	88
*cac*^*TS2*^*/Y; sda* (HS)	0	100
*eas cac*^*TS2*^*/Y* (RT)	90	10
*eas cac*^*TS2*^*/Y* (HS)	54	46
*para*^*bss1*^ *cac*^*TS2*^*/Y* (RT)	36	64
*para*^*bss1*^ *cac*^*TS2*^*/Y* (HS)	13	87
*para*^*bss1*^ *cac*^*TS2*^ (RT)	23	77
*para*^*bss1*^ *cac*^*TS2*^ (HS)	8	92
*para*^*bss1*^ *cac*^*TS2*^*/para*^*+*^ *cac*^*TS2*^ (RT)	2	98
*elav*^*c155*^*-GAL4 para*^*bss1*^*/Y;UAS-cacRNAi*/+) (RT)	64	36
*elav*^*c155*^*-Gal4 eas/Y;UAS-cacRNAi/+*) (RT)	15	85

At restrictive temperatures, *cac*^*TS2*^ exhibits complex phenotypes including TS seizure-like activity, synaptic failure and paralysis. We found that all *cac*^*TS2*^ phenotypes are reciprocally suppressed in double mutant combination with *para*^*bss1*^. Suppression of TS seizure-like behaviors in *cac*^*TS2*^ by a Na^+^ channel mutation indicates that the combination of two ion channel alleles involved in epilepsy can have beneficial clinical effects when present in the same individual organism: that is, each of the two mutations co-suppresses seizures caused by the other, similar to observations reported for mouse [17].

## Results

### The *cac*^*TS2*^ mutant is seizure-resistant at room temperature; seizure-sensitive at high temperature

The behaviors of *cac*^*TS2*^ mutants are unexceptional at room temperature (24°C): feeding, grooming, and mating behaviors appear normal. Overall activity levels are unaltered: flies are neither sluggish nor hyperactive. The *cac*^*TS2*^ mutants show no bang-sensitive (BS) behavioral paralysis phenotype and are unaffected by mechanical stimulation. Using the adult giant fiber (GF) neurocircuit as a proxy for holo-nervous system function, the electrophysiology phenotype for *cac*^*TS2*^ at room temperature generally resembles wild-type [18]. Thus, single pulse stimulation of the GF produces evoked potentials and synaptic currents in the dorsal longitudinal muscle (DLM) that are normal in appearance (S1 Fig) [5], have a threshold of 0.96 ± 0.12 V (mean ± s.e.m., n = 9) and a latency of 1.1 ± 0.04 msec (mean ± s.e.m., n = 5).

**Fig 1:**
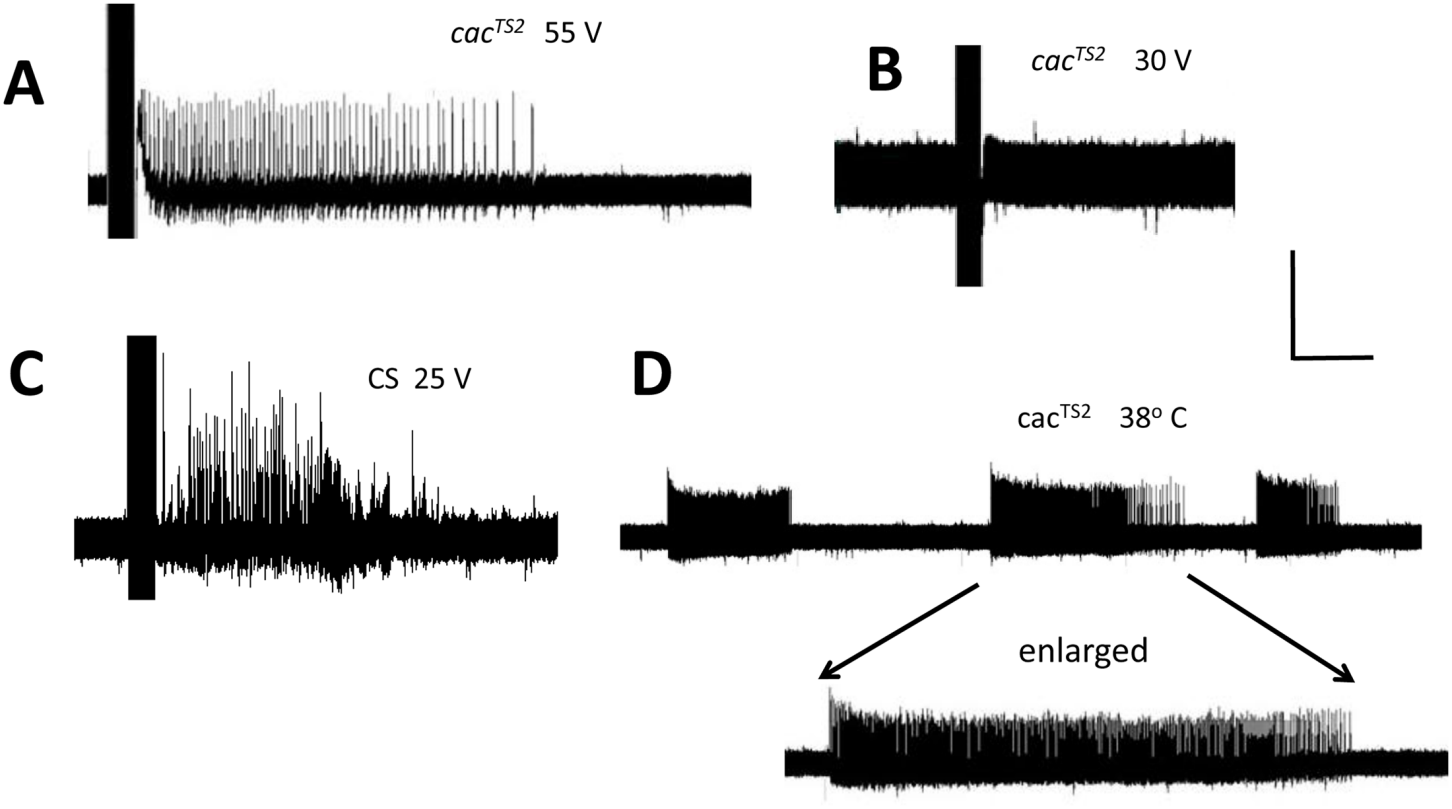
*Drosophila cac*^*TS2*^ electrophysiology. A. Electrical recording from a *cac*^*TS2*^ DLM fiber following delivery of 55 V HFS stimulation (0.5 msec stimuli at 200 Hz for 300 msec) at room temperature. The stimulation evokes seizure-like electrical activity, indicating that the voltage is at or above seizure threshold. B. Recording from a *cac*^*TS2*^ DLM fiber following delivery of a 30 V HFS stimulus showing that this stimulation voltage fails to evoke seizure-like activity. This stimulation voltage, near the wild-range, is below seizure threshold for *cac*^*TS2*^. C. Recording from a wild type Canton Special (CS) DLM fiber following delivery of a 25 V HFS stimulus that is effective in evoking seizure-like activity. D. Recording from a *cac*^*TS2*^ DLM fiber following a temperature shift from permissive room temperature to restrictive 38°C temperature. Spontaneous seizure-like electrical activity is observed in the DLM fiber, indicating that the mutant is seizure-sensitive at restrictive temperatures. Recording shows a representative example of three spontaneous seizure-like discharges. Horizontal calibration is 1.0 sec for A, B, and C; 4 sec for D and 1.5 sec for D (enlarged). Vertical calibration is 20 mV.

Seizure-like electrical activity in *cac*^*TS2*^ mutants can be evoked with high-frequency stimuli (HFS; 0.5 msec stimuli at 200 Hz for 300 msec; Fig 1A), similar to discharges observed for other *Drosophila* mutants [19,20]. Large HFS voltages were characteristically required to evoke seizures at room temperature indicating that *cac*^*TS2*^ behaves as a seizure-resistant mutant. Seizure threshold for *cac*^*TS2*^ was (58.3 ± 1.0 V HFS, mean ± s.e.m., n = 13), nearly twice that of Canton-Special wild type flies (24.64 ± 2.83 HFS, mean ± s.e.m., n = 10; Fig 1A–1C). The seizure threshold for *cac*^*TS2*^ is comparable to previously-reported seizure-resistant mutants such as *paralytic*^*ts1*^, *Shaker*^*KS133*^ and *shakingB*^*2*^ that have high seizure thresholds and can also act as seizure-suppressor mutations in double mutant combinations with BS mutants [19].

Several *cac* alleles, especially *cac*^*TS2*^ and *cac*^*NT27*^, are notable for their temperature-sensitivity (TS) with essentially normal behavior and neurology at room temperature which is permissive; and displaying complicated neurological phenotypes at high temperature (>38°C) which is restrictive [5,8]. The shift from permissive to restrictive temperature, causes a transient period of nervous system hyperexcitability lasting several seconds [8], followed by a prolonged period of hypoexcitability with synaptic failure and behavioral paralysis [5]. The hyperactive period is characterized by spontaneous seizure-like behaviors: leg-shaking, abdominal twitching, wing scissoring, and proboscis extensions. These are accompanied by spontaneous seizure-like firing of the DLM motor neurons in electrophysiology recordings (Fig 1D), similar to that described previously [8]. Seizure-like activity for *cac*^*TS2*^ at elevated temperature is interesting considering the seizure-resistant phenotype observed at room temperature. The spontaneous seizure-like DLM activity generally resembles that observed during evoked seizure-like activity (Fig 1). We were unable to determine a reliable evoked seizure-threshold for *cac*^*TS2*^ at restrictive temperature. Immediately following the shift to restrictive temperature, the seizure-threshold is still high, resembling the threshold of *cac*^*TS2*^ at room temperature. Spontaneous seizure-like activity ensued soon after the shift to restrictive temperature and their nearly continuous occurences made it difficult to distinguish them from stimulus-evoked seizures. Taken together, these findings indicate that *cac*^*TS2*^ is apparently a seizure-resistant mutant at room temperature, changing to a seizure-sensitive mutant after shift to restrictive temperature.

### The *cac*^*TS2*^ mutation suppresses BS paralysis in *sda* and *eas* mutants

**Fig 2:**
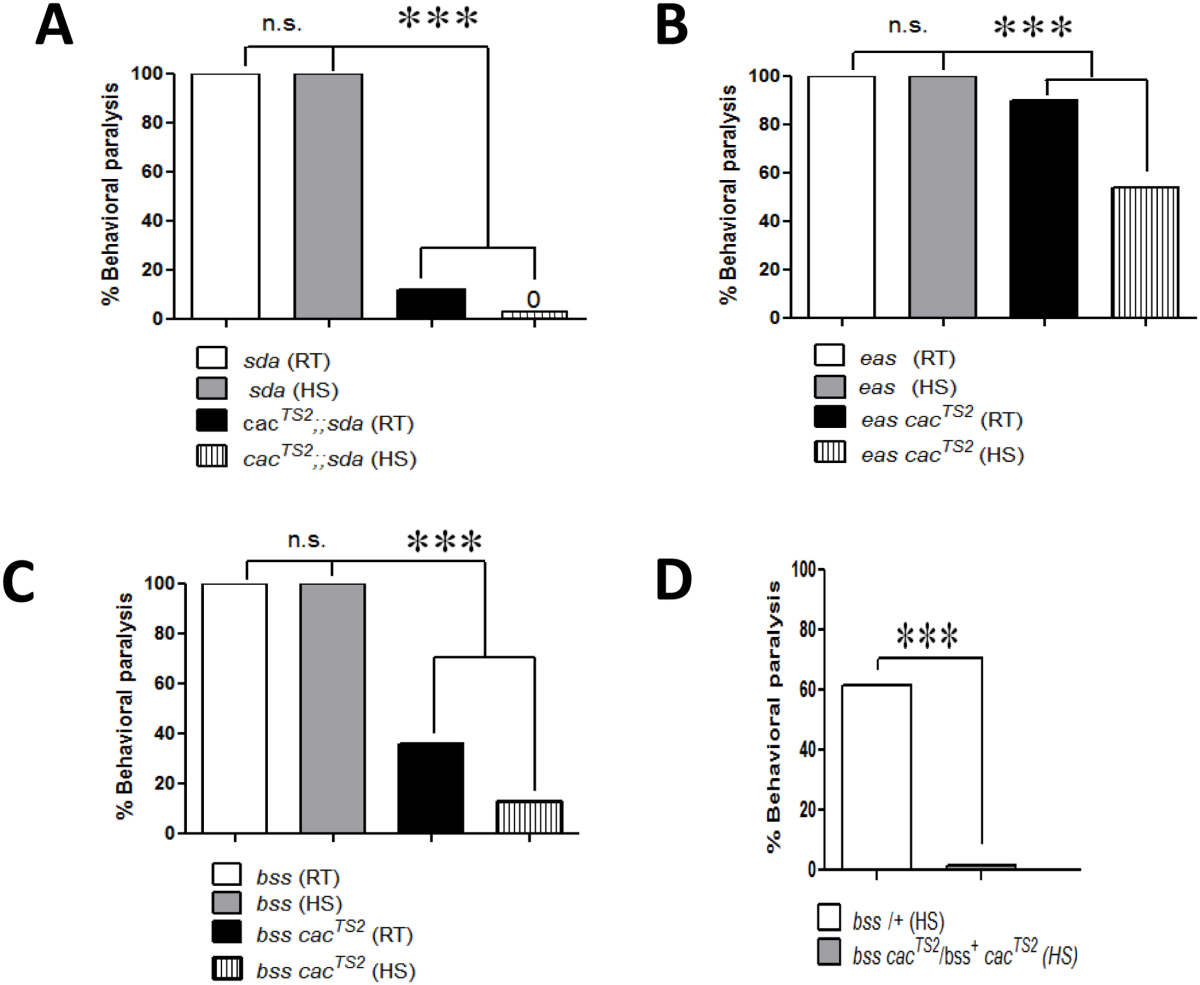
Suppression of bang sensitive behavioral phenotypes by *cac*^*TS2*^. A. Behavioral paralysis in *sda* hemizygotes (white bar) is suppressed in hemizygous double mutants *cac*^*TS2*^*/Y;;sda* at room temperature (RT) by about a factor of 10 (black bar; n = 147). Following a brief heat shock (HS = 3 min at 30°C), complete suppression of behavioral paralysis is observed in hemizygous double mutants *cac*^*TS2*^*/Y;;sda* (striped bar; n = 93). B. Behavioral paralysis in hemizygous *eas cac*^*TS2*^*/Y* double mutants at RT (black bar, n = 147) shows a small reduction compared to hemizygous *eas/Y* (white bar). Following a brief heat shock (HS = 3 min at 30°C), suppression is by about a factor of 2 (striped bar; n = 93). C. Behavioral paralysis in *para*^*bss1*^ hemizygotes (white bar) is suppressed in hemizygous *para*^*bss1*^ *cac*^*TS2*^ double mutants at RT by about a factor of 3 (black bar; 64% suppression; n = 658). Following a brief HS, suppression is increased in the *para*^*bss1*^ *cac*^*TS2*^ double mutants (striped bar; 87% suppression; n = 650). D. Following a brief HS, *cac*^*TS2*^ completely suppresses behavioral paralysis in *para*^*bss1*^*/+* heterozygous females (n = 60). *P < 0.01, **P = 0.001, ***P < 0.0001, chi-square test.

In order to test for genetic interactions, we constructed double mutants between *cac*^*TS2*^ and different BS mutants. This resulted in suppression of the BS phenotype as exemplified by hemizygous double mutant male flies *cac*^*TS2*^*/Y;;sda* that showed 12% behavioral BS paralysis (indicating 88% phenotypic suppression, n = 147; P < 0.0001, chi- square test; Fig 2A; Table 1) at room temperature (24°C), compared to the *sda* single mutant control flies which showed 100% BS paralysis (P < 0.0001). This finding of suppression at room temperature was a little unexpected since *cac*^*TS2*^ has previously been described as a temperature-sensitive mutation and permissive temperature phenotypes have not been reported. This result may be related to the observation above that *cac*^*TS2*^ is seizure-resistant at room temperature; heretofore the only other difference from wild type that we have seen at room temperature.

In order to examine if temperature has an effect on BS suppression, we examined double mutants at elevated temperatures within the nominally permissive range, that is below 38°C, to avoid *cac*^*TS2*^ behavioral paralysis. We found that seizure-suppression by *cac*^*TS2*^ is increased at elevated temperatures. In hemizygous double mutant flies *cac*^*TS2*^*/Y;;sda*, a brief heat shock (HS; 3 min at 30°C), completely suppressed all *sda* bang-sensitivity (0% paralysis, 100% suppression, n = 93; P < 0.0001, chi-square test; Fig 2A; Table 1). A similar brief HS delivered to control single mutant *sda* flies had no effect on bang-sensitivity: 100% of control flies continued to show BS paralysis (P < 0.0001).

The *cac*^*TS2*^ mutation is a general seizure-suppressor, not limited to suppression of BS phenotypes in *sda* mutants: modest seizure-suppression is also observed for *eas* mutants. In hemizygous double mutant male flies *eas cac*^*TS2*^*/Y*, BS was 90% (10% suppression, n = 147; P = 0.0002, chi- square test; Fig 2B; Table 1) at room temperature compared to 100% BS in *eas* single mutant controls. In *eas*, suppression by *cac*^*TS2*^ was also increased with exposure to elevated temperature. In hemizygous double mutant flies *eas cac*^*TS2*^*/Y*, bang-sensitivity was 54% (46% suppression, n = 93; P = < 0.0001, chi-square test; Fig 2B; Table 1) following a brief HS (3 min at 30°C). HS had no effect on the bang sensitivity of *eas* single mutant control flies: 100% of the control flies showed BS paralysis (P < 0.0001). Thus, *cac*^*TS2*^ acts as a general suppressor of BS behavior, reverting phenotypes of both *sda* and *eas* BS mutants in double mutant combinations. Some suppression occurs at room temperature, although suppression increases with increases in temperature within the permissive temperature range. Previous studies have also shown that BS phenotypes in *sda* mutants are easier to suppress than for *eas* mutants [21,22].

### The *cac*^*TS2*^ mutation suppresses BS paralysis in *para*^*bss1*^ mutants

We investigated genetic interactions between *cac*^*TS2*^ and *para*^*bss1*^ by constructing the appropriate double mutant combinations. Previous studies have found that seizure-like phenotypes are difficult to suppress in *para*^*bss1*^ mutants, that carry a gain-of-function voltage-gated Na^+^ channel defect [10 (Table 2)]. We find here that *cac*^*TS2*^ is an effective suppressor of *para*^*bss1*^ behavioral phenotypes. For hemizygous double mutant males (genotype: *para*^*bss1*^ *cac*^*TS2*^*/Y*), BS paralysis was 36% (64% suppression; n = 658; P < 0.0001, chi-square test) at room temperature (Fig 2C; Table 1). Suppression by *cac*^*TS2*^ was also increased at elevated temperature. After a brief HS, BS paralysis decreased to 13% (87% suppression; n = 650; P < 0.0001, chi-square test) in hemizygous double mutants (Fig 2C; Table 2). Homozygous double mutant females (genotype: *para*^*bss1*^ *cac*^*TS2*^) showed 23% BS paralysis at room temperature (77% suppression) which decreased to 8% (92% suppression) following HS (Table 2). In control *para*^*bss1*^ flies, there was no effect of HS: 100% of flies showed BS paralysis (P < 0.0001; Table 2). The *cac*^*TS2*^ mutation is an especially effective suppressor of *para*^*bss1*^*/+* heterozygote behavioral phenotypes. In double mutant flies (genotype: *para*^*bss1*^ *cac*^*TS2*^*/para*^*+*^ *cac*^*TS2*^) BS paralysis was 2% (98% suppression) at room temperature, compared to 62% BS paralysis seen in control *para*^*bss1*^*/+* heterozygotes without the *cac*^*TS2*^ suppressor (n = 60; P < 0.0001, chi-square test; Fig 2D; Table 2).

**Table 2:** Seizure suppression of *para*^*bss1*^ by *cac*^*TS2*^ and other suppressors. The table lists four mutations that have been reported to revert *para*^*bss1*^ phenotypes in double mutant combinations. An additional 12 seizure-suppressor mutations have been reported that are ineffective at suppressing *para*^*bss1*^ [22].

Suppressor	Genotype	Threshold (HFS V)	Effect	Reference
*cac*^*TS2*^	*para*^*bss1*^ *cac*^*TS2*^	51.6 ± 1.2	Homozygous, hemizyous, and heterozygous *para*^*bss1*^ flies are all suppressed.	Current paper
*mle*^*napts*^	*para*^*bss1*^*; mle*^*napts*^	29 ± 4.7	Suppresses homozygous *para*^*bss1*^ flies	13, 14
*shi*^*ts1*^	*shi*^*ts1*^ *para*^*bss1*^	3.5 HFS V	Behavioral suppression at elevated temperatures. Minimal effect on seizure threshold.	15
*gish*	*para*^*bss1*^*/+;gish*^*04895*^*/+*	15.6 ± 2.4	Specific supppressor of heterozygous *para*^*bss1*^*/+* flies. Does not suppress *para*^*bss1*^ homozygotes and hemizygotes. Does not suppress *sda* or *eas*.	16

The salient consequence of *cac*^*TS2*^ suppression is the increased percentage of flies escaping BS paralysis, but flies that undergo paralysis are also influenced by the suppressor: observed as a reduction in the time required for recovery. Control *para*^*bss1*^*/Y* mutant males when paralyzed ordinarily have a long recovery time 195 sec. In contrast, paralyzed flies carrying the suppressor (genotype: *para*^*bss1*^ *cac*^*TS2*^*/Y*) have about a four-fold reduction in the time to recovery 46 sec (n = 45; P < 0.0001, unpaired student t-test). Moreover, *cac*^*TS2*^ reduced the refractory time period for hemizygous double mutant male flies *para*^*bss1*^ *cac*^*TS2*^*/Y*. Double mutants show a shorter refractory time period of 17 min, compared to 25 min for *para*^*bss1*^ single mutant flies (n = 34; P = 0.0005, unpaired student t-test).

### The electrophysiology of *cac*^*TS2*^ seizure-suppression

**Fig 3:**
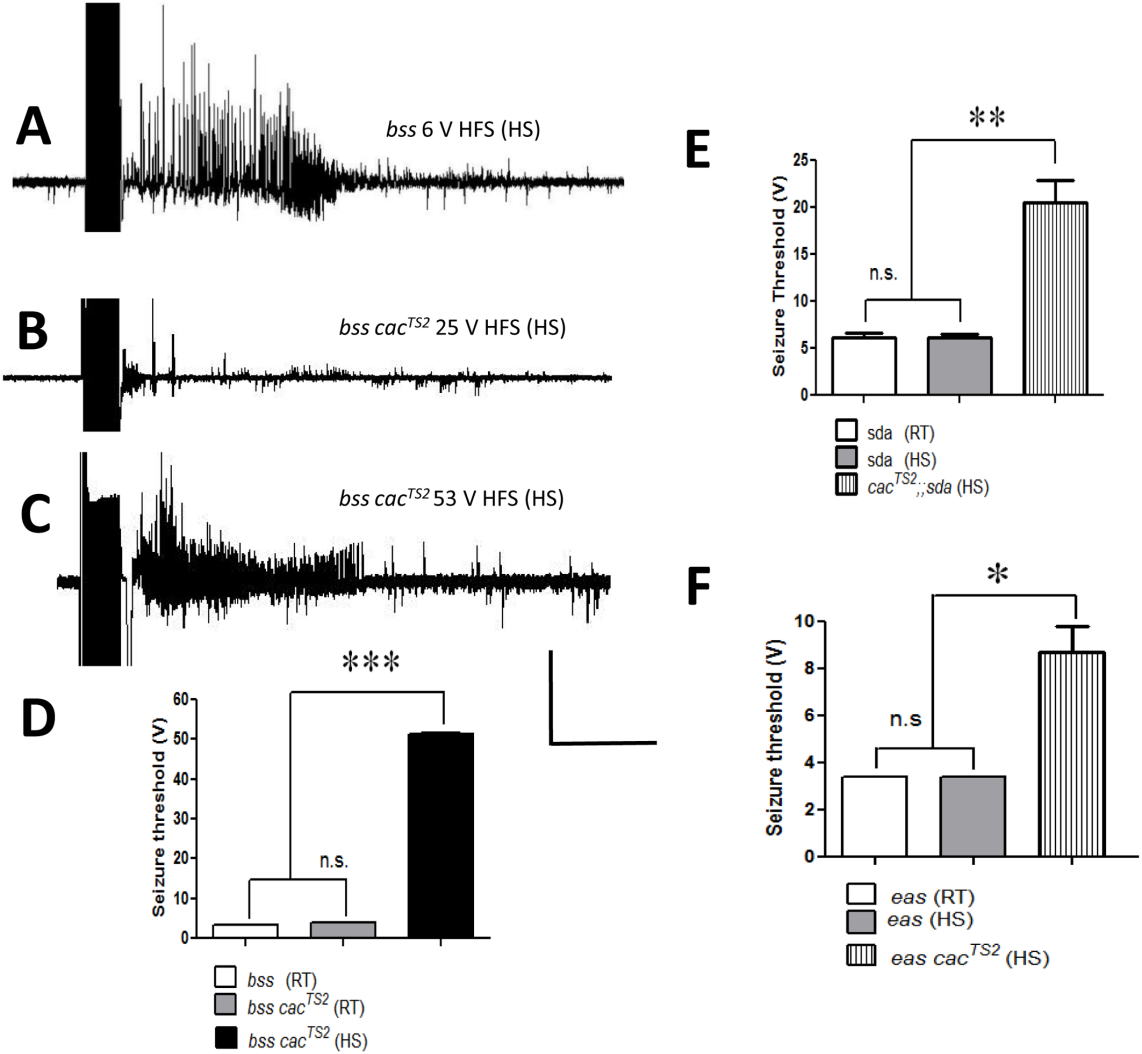
Electrophysiology of *cac*^*TS2*^ suppression. A. Electrical recording from a *para*^*bss1*^ DLM fiber showing seizure-like activity evoked by a 6 V HFS stimulation, showing that the single BS mutant has a low seizure threshold. B. Electrical recording from a *para*^*bss1*^ *cac*^*TS2*^ DLM fiber showing that stimulation at 25 V HFS is below threshold and fails to evoke a seizure event in the double mutant indicating suppression by *cac*^*TS2*^. C. Electrical recording from a *para*^*bss1*^ *cac*^*TS2*^ DLM fiber showing that a high voltage stimulation at 53 V HFS is above threshold and evokes a seizure-like event in the double mutant. For the traces depicted in A-C, the HFS stimulus is delivered immediately after HS (3 min at 30°C). D. At room temperature (RT; steady state), seizure threshold for the *para*^*bss1*^ *cac*^*TS2*^*/Y* double mutant (gray bar) is similar to the *para*^*bss1*^*/Y* single mutant (white bar). Immediately following a HS, seizure threshold for *para*^*bss1*^ *cac*^*TS2*^*/Y* is transiently high indicating suppression (black bar; n = 7). E. Average seizure threshold is increased by about a factor of 4 in *cac*^*TS2*^*/Y;;sda* double mutants following a brief HS (striped bar; 20.5 ± 2.42 V HFS; n = 7) compared to hemizygous *sda* single mutant (white and gray bars). F. Average seizure threshold is increased by about a factor of 3 in hemizygous double mutant *eas cac*^*TS2*^*/*Y following a brief HS (striped bar; 8.7 ± 1.1 V HFS; n = 11) compared to the *eas* single mutant (white and gray bars). Quantitative data are represented as mean ± s.e.m. *P < 0.01, **P = 0.001, ***P < 0.0001, (D-F) chi-square test. Horizontal calibration: 800 msec for A-C; Vertical calibration: 20 mV for A-C.

Seizure suppression by *cac*^*TS2*^ is also observed in evoked seizure-like neuronal activity recorded electrophysiologically. This analysis shows an unusual seizure-suppression of *para*^*bss1*^ by *cac*^*TS2*^. Immediately following HS, there is considerable suppression of *para*^*bss1*^ (Fig 3A–3D), but this suppression is transient and short-lasting (Fig 3D). Thus, immediately following the HS (3 min at 30°C), seizure-threshold for *para*^*bss1*^ *cac*^*TS2*^ double mutants is high (51.6 ± 1.2 V HFS, mean ± s.e.m., n = 7; P < 0.0001, ANOVA test; Fig 3D). This is greater than the seizure threshold for the *para*^*bss1*^ by about a factor of ten, and greater than wild type seizure-threshold, by nearly a factor of two; flies with seizure-thresholds in this range are seizure-resistant mutants (Table 3) [14]. Seizure-threshold quickly decreases when maintained at room temperature and the steady-state seizure-threshold of *para*^*bss1*^ *cac*^*TS2*^ double mutants at room temperature is (3.82 ± 0.2 V HFS, mean ± s.e.m., n = 10), similar to the *para*^*bss1*^ single mutant (3.2 ± 0.1 V HFS, mean ± s.e.m., n = 8; Fig 3A). The time course of the threshold change is difficult for us to determine with our present electrophysiology protocols, but it appears similar to changes in BS behavior following HS, about 5–7 min.

**Table 3:** Seizure thresholds for different single mutant and double mutant genotypes. Seizure-like activity is evoked by high frequency (HF) stimuli delivered to the brain (0.5 ms pulses at 200 Hz for 300 ms). Stimulation voltage was gradually increased and seizure threshold, a measure of seizure-susceptibility, was defined as the minimum voltage required for HF stimulation to become an electroconvulsive shock; that is, to induce seizure activity. Each genotype has a characteristic seizure threshold. Values of seizure threshold are presented as mean V HFS ± s. e. m. with n ≥ 5.

Genotype	Threshold (V HFS)
*bss* (RT)	3.2 ± 0.10
*bss* (HS)	6.9 ± 0.35
*eas/Y* (RT)	3.8 ± 0.11
*eas/Y* (HS)	3.7 ± 0.11
*sda* (RT)	6.2 ± 0.30
*sda* (HS)	6.8 ± 0.23
CS/Y (RT)	24.6 ± 2.83
CS/Y (HS)	24.3 ± 3.9
*eas cac*^*TS2*^*/Y*(HS)	8.7 ± 1.12
*cac*^*TS2*^*;;sda* (HS)	20.5 ± 2.42
*bss cac*^*TS2*^ (RT)	3.8 ± 0.2
*bss cac*^*TS2*^ (HS)	51.6 ± 1.22
*cac*^*TS2*^ (RT)	58.3 ± 1.00

Electrophysiology analysis also shows *cac*^*TS2*^ suppression of other BS mutants. The *cac*^*TS2*^*;;sda* double mutant has a seizure-threshold of (20.5 ± 2.42 V HFS, mean ± s.e.m., n = 7) following HS and tested at room temperature, about 3-fold higher than the threshold of the *sda* single mutant (6.2 ± 0.3 V HFS, mean ± s.e.m., n = 5, P < 0.0001, unpaired student t-test; Fig 3E). That is, in the double mutant, there is a reversion of BS electrophysiology by *cac*^*TS2*^ to nearly the wild-type range of seizure-threshold. The *cac*^*TS2*^ mutation also suppresses *eas* seizure-like activity. Hemizygous double mutant flies *eas cac*^*TS2*^*/*Y have a seizure-threshold of (8.7 ± 1.1 V HFS, mean ± s.e.m., n = 11) following HS, about two-fold higher than the low threshold of the *eas* single mutant (3.8 ± 0.1 V HFS, mean ± s.e.m., n = 6; P < 0.01; unpaired student t-test; Fig 3D; Table 3).

### Seizure-suppression by cacRNAi

**Fig 4:**
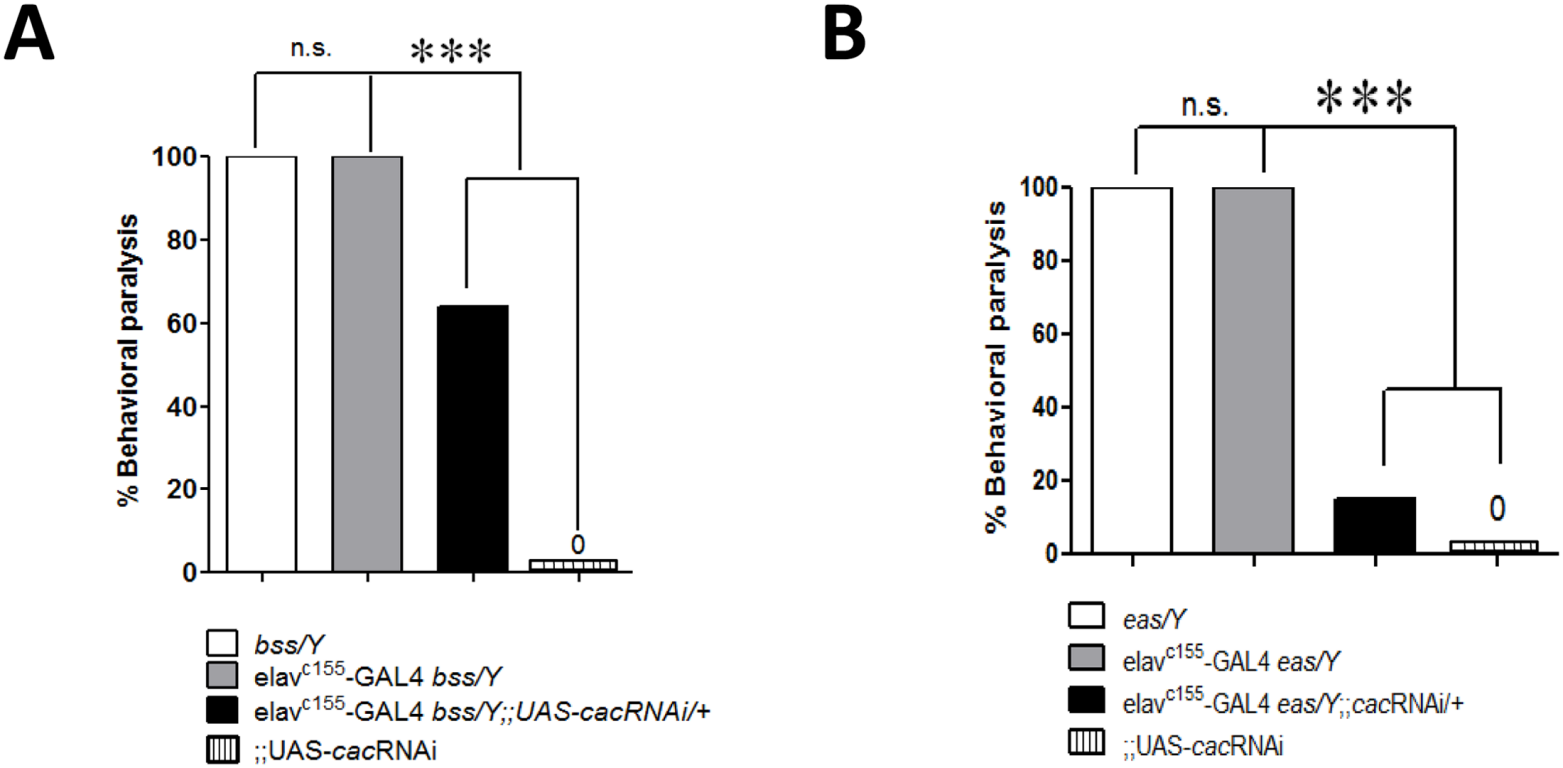
Suppression of *para*^*bss1*^ and *eas* behavioral phenotypes by *cac*RNAi at room temperature. A. Behavioral paralysis in *para*^*bss1*^ hemizygotes (white bar) is suppressed by *cac*RNAi when expressed by a pan-neuronal driver (black bar; genotype: *para*^*bss1*^ *elav*^*c155*^*-GAL4/Y;;UAS-cacRNAi*). B. Behavioral paralysis in *eas* hemizygotes (white bar) is suppressed by *cac*RNAi when expressed with a pan-neuronal driver (black bar; genotype: *eas elav*^*c155*^*-GAL4/Y;;UAS-cacRNAi*). ***P < 0.0001, chi-square test (n = 212 and 74 for panels A and B, respectively).

To further study *cac* seizure-suppression, we generated loss-of-function *cac* genotypes using *cacRNAi* to knockdown *cac* expression; these were tested for BS mutant suppression. Flies utilizing a pan-neuronal GAL4 driver and one copy of *cacRNAi* were viable and had largely normal behavior. Similar to the *cac*^*TS2*^ mutation, *cacRNAi* suppressed BS behavioral phenotypes in double mutant male *para*^*bss1*^ flies. Males (genotype: *elav*^*c155*^*-GAL4 para*^*bss1*^*/Y;;UAS-cacRNAi*/+) showed BS paralysis in 64% of flies (36% suppression; n = 212; P < 0.0001, chi-square test; Fig 4A) at room temperature. Suppression of BS by *cacRNAi* was more effective in *eas* mutants. Males (genotype: *elav*^*c155*^*-Gal4 eas/Y;;UAS-cacRNAi/+*) showed BS paralysis in 15% of flies (85% suppression, n = 74; P < 0.0001, chi- square test; Fig 4B). Although cacRNAi was effective at suppressing BS phenotypes, in other respects it was different than *cac*^*TS2*^ mutations because it did not cause temperature-sensitive phenotypes. Thus, male cacRNAi flies (genotype: *elav*^*c155*^*-GAL4/Y;;UAS-cacRNAi*/+) at 38°C showed netiher spontaneous seizure-like behaviors no behavioral paralysis.

### Suppression of *cac*^*TS2*^ seizure phenotypes by the *para*^*bss1*^ mutation

**Fig 5:**
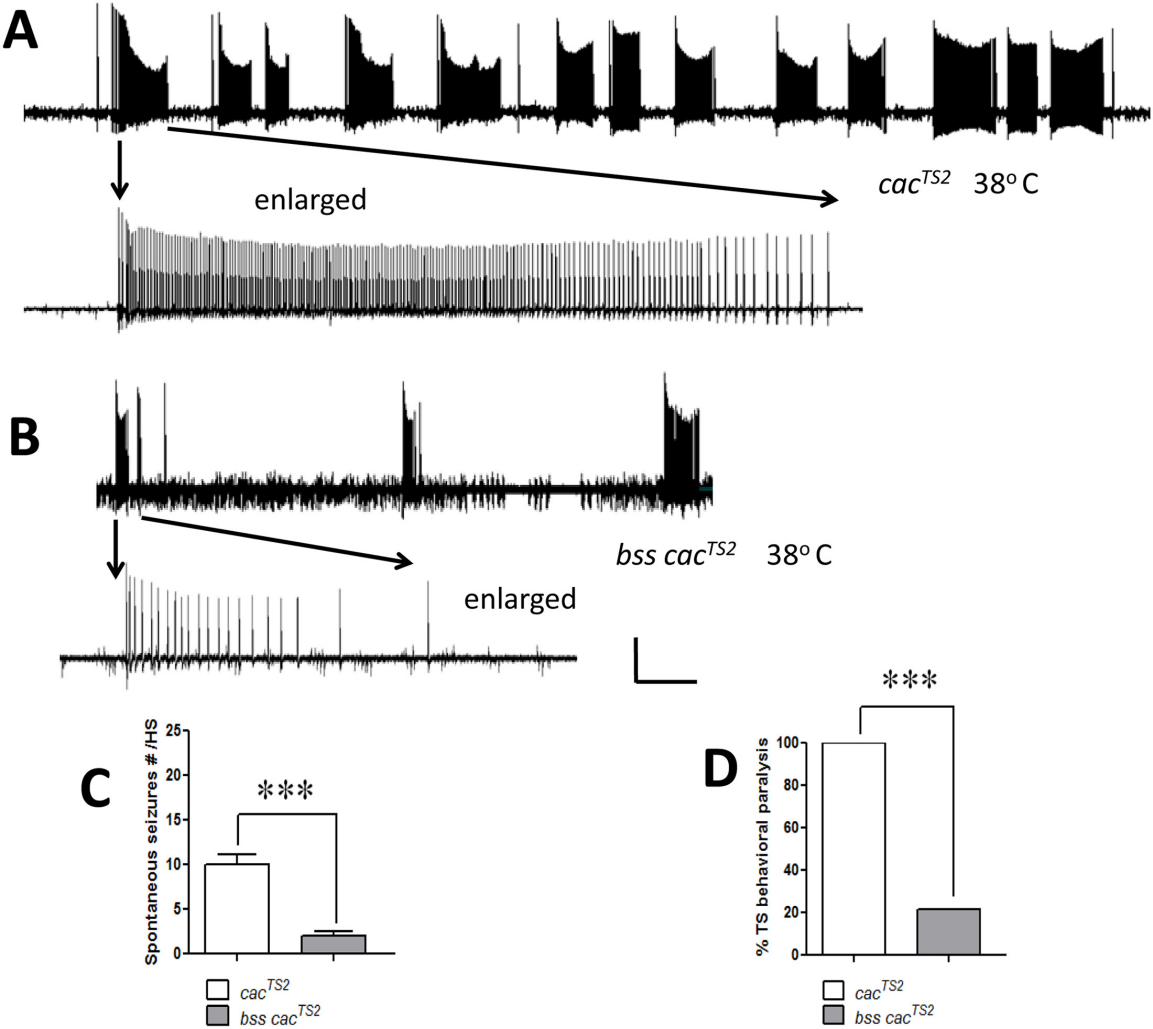
Suppression of *cac*^*TS2*^ temperature-sensitive seizure-like activity by *para*^*bss1*^. A. Spontaneous seizure-like activity observed in a *cac*^*TS2*^ single mutant when the temperature is increased from room temperature to 38°C, indicating that the mutant is seizure-sensitive at restrictive temperature. Recording shows a representative example of 13 spontaneous seizure-like discharges during HS. Enlargement (lower trace) shows one of the spontaneous discharges at a higher sweep speed. B. Spontaneous seizure-like activity is decreased in double mutant homozygous *para*^*bss1*^ *cac*^*TS2*^ at restrictive temperature. C. Number of spontaneous seizure-like activity evoked due to restrictive temperature and measured by electrophysiology. Compared to *cac*^*TS2*^, in homozygous *para*^*bss1*^ *cac*^*TS2*^ double mutant flies number of TS seizure-like activity is reduced by about a factor of 5 (white bar: 10 ± 1.2 events/3 min HS; n = 10; compared to gray bar: 2.5 ± 0.42 events/3 min HS; n = 20). D. Behavioral seizure-like activity is decreased in homozygous double mutant *para*^*bss1*^ *cac*^*TS2*^ flies at restrictive temperature. Compared *to cac*^*TS2*^ single mutants (white bar), *para*^*bss1*^ *cac*^*TS2*^ double mutants (gray bar) exhibit 5 fold decrease in TS seizure-like behaviors. Quantitative data are represented as mean ± s.e.m. ***P < 0.0001, based on unpaired student-t test (C) and chi-square test (D). Horizontal calibration: A. 15 sec, A (enlarged) 1 sec, B. 15 sec, B (enlarged) 1.5 sec. Vertical calibration: 10 mV.

Double mutant *para*^*bss1*^ *cac*^*TS2*^ flies were examined following a temperature shift from room temperature to 38°C, the restrictive temperature for *cac*^*TS2*^. Interestingly, some temperature-sensitive phenotypes, prominent in the *cac*^*TS2*^ single mutant, were reduced in the double mutant, apparently suppressed by the presence of *para*^*bss1*^ in the double mutant combination. In *para*^*bss1*^ *cac*^*TS2*^ flies the TS spontaneous seizure-like electrophysiological phenotype was greatly reduced (Fig 5A and 5B). Electrophysiological recordings from *cac*^*TS2*^ single mutants show the number of spontaneous seizure-like discharges was 10 ± 1 spontaneous discharges/HS (mean ± s.e.m., n = 10; HS = 3 min at 38°C. Fig 5A and 5C). In contrast, recordings from *para*^*bss1*^ *cac*^*TS2*^ double mutants show 2.5 ± 0.42 spontaneous discharges/HS (mean ± s.e.m., n = 20, P = 0.0003, unpaired student-t test; Fig 5B and 5C). In addition to the number of spontaneous discharges being reduced, there also appeared to be a reduction in discharge duration (Fig 5A and 5B). The temperature-sensitive behavioral paralysis phenotype of *cac*^*TS2*^ was also suppressed by *para*^*bss1*^ (Fig 5D). For the *cac*^*TS2*^ single mutant, 100% of flies undergo paralysis when the temperature is increased from room temperature to 38°C, as described in previously [5]. In contrast, for *para*^*bss1*^ *cac*^*TS2*^ double mutants, only 20% of flies are paralyzed at 38°C (80% suppression, n = 76; P < 0.001, chi-square test, Fig 5D).

## Discussion

We find that *cac*^*TS2*^ is a general seizure-suppressor mutation, reverting neurological phenotypes of several BS mutants: *sda*, *eas*, and *para*^*bss1*^. Suppression of *para*^*bss1*^ is especially notable because it is a BS mutant that has previously been difficult to modify by suppressor mutation [21,22] or antiepileptic drug [23–27]. Recently, directed efforts to target *para*^*bss1*^ by suppressors have identified two: *gilgamesh (gish)* and *shibire*^*ts*^ *(shi*^*ts*^*)*, although both appear somewhat weaker than *cac*^*TS2*^ [15–16 (Table 1)]. Suppression by *gish* is unusual because it is selectively effective against *para*^*bss1*^*/+* heterozygotes; *gish* does not suppress homozygous *para*^*bss1*^. Also, *gish* does not suppress other BS mutants, such as *sda* or *eas*. [16]. For *shi*^*ts*^, suppression of *para*^*bss1*^ is not evident at room temperature, but occurs with increased temperature that causes interference with endocytosis during synaptic vesicle recycling [15]. BS suppression reported here for *cac*^*TS2*^ is comparable or better than for *gish* and *shi*^*ts*^.

The major questions arising from this study are: how does *cac*^*TS2*^ suppression work? And what is it about the *cac*^*TS2*^ mutation that makes it such an effective suppressor of *para*^*bss1*^ primary phenotypes, BS behavior and seizure threshold? A complete answer to these questions remains unclear from the experiments we are able to perform here, but leads to speculation about mechanisms of seizure, and about how seizure-suppression might be accomplished. The *cac*^*TS2*^ allele behaves as a recessive loss-of-function mutation with reduced neurotransmitter release at the neuromuscular junction and paralysis at high temperature [5]. Also at high temperature, the mutant displays considerable spontaneous seizure-like activity seen in muscle fiber recordings [8]. At first, it might appear that this seizure-like activity is inconsistent with the *cac*^*TS2*^ phenotype of reduced transmitter release, especially if this reflects an overall reduction in excitability. The *cac*^*TS2*^ seizure-like activity must be due to spontaneous action potential bursting in adult and larval motor neurons; the activity recorded in the muscle fiber is reflecting seizure-like motor neuron firing. We suggest that this motor neuron firing may be due to a loss of inhibitory synaptic activity impinging on them, possibly causing some type of post-inhibitory rebound excitation within the motor neurons. That is, as excitatory transmitter reduction by temperature is observed at the neuromuscular junction, synaptic inhibition that ordinarily limits motor neuron firing is concurrently reduced leading to spontaneous seizure-like activity observed in muscle.

About fifteen mutations have been identified previously as seizure-suppressors [reviewed in 22]. Some of these suppressors encode well-studied gene products that have not heretofore been associated with neuronal signaling or membrane excitability such as the de-ubiquitinase USP9X [28] and DNA topoisomerase I [29]. Some of the seizure-suppressor genes encode neuronal signaling molecules that have allowed us to consider previously three likely mechanisms for how seizure-like activity might be suppressed by second-site mutations; here, suppression by *cac*^*TS2*^ suggests to us a fourth mechanism. Previously, we found that:

1. Seizure-suppression can occur by limiting high frequency action potential firing. The K^+^ channel mutation *Sh*^*KS133*^, increases action potential duration, leading to long refractory periods [14,19,30]. Action potentials cannot be generated during the refractory period, apparently leading to seizure-suppression by limiting axons to low action potential firing frequencies. Thus, despite *Sh*^*KS133*^ generally causing nervous system hyperexcitability, the mutation is also a seizure-suppressor because the high-frequency action potential firing required for seizure-like activity is not supported by axons in these mutants.
2. Seizure-suppression can occur by opposing effects on nerve excitability [9,14,19,31,32]. The *para*^*ST76*^, *para*^*JS*^ and *mle*^*napts*^ mutations reduce nerve excitability by Na^+^ channel loss-of-function. Hypoexcitability from these mutations can suppress seizure-like activity in BS mutations causing hyperexcitability such as *eas* and *sda*.
3. Seizure-suppression can occur by preventing synchronized firing. The *ShakB*^*2*^, a mutation of the gap junction innexin channel, causes a defect in electrical synaptic transmission. Seizure-like activity is suppressed because electrical synaptic transmission appears to be critical for synchronizing the activity of populations of firing neurons and for the spread of seizure-like excitation [14,33].

The signaling molecules responsible for the process of chemical synaptic transmission are a potentially rich source for identifying seizure-suppressor mutations. Seizure-suppressors are most logically expected from among mutations enhancing inhibitory GABAergic synaptic transmission or mutations diminishing excitatory cholinergic transmission. Some other mutations are most logically expected to enhance seizure phenotypes such as mutations decreasing GABAergic function or enhancing cholinergic transmission. It is more difficult to anticipate the effect on seizures of mutations affecting general synaptic transmission properties, that is, molecules that are common to both excitatory and inhibitory synaptic processes. The *cac*^*TS2*^ mutation examined here is such a mutation, the *cac* gene encodes the α_1_, primary structural subunit of the voltage-gated Ca^++^ channel responsible for triggering regulated synaptic vesicle release at both excitatory and inhibitory synapses [4,34]. Thus, it was surprising that *cac*^*TS2*^ was not only a seizure-suppressor mutation, it was one of the most effective suppressors that we have identified. Because of this, we propose that *cac*^*TS2*^ suppression may work via a somewhat different mechanism than we have observed previously, generally, using neurocircuitry to cause seizure suppression. We presume that the suppression works by interfering with chemical synaptic transmission in many or most circuits in the fly. Modest interference in synaptic transmission at room temperature is sufficient to suppress weak BS mutants, such as *sda*. Stronger disabling of synaptic transmission following a heat pulse is necessary to suppress the stronger BS *para*^*bss1*^.

We thought it possible to identify specific circuits responsible for suppression by the differential GAL4/UAS expression of cacRNAi. Our initial attempts expressed cacRNAi selectively in excitatory interneurons (cha-GAL4 driver), or inhibitory interneurons (GAD-GAL4 driver). Expression of cacRNAi in different interneuronal populations was a little less effective than pan-neuronal expression, but differences were small (S2 Fig). From this limited investigation, we do not find indications for specific circuits suppressing seizures or, if they exist, how we might go about discovering them. We entertain the interesting possibility that *cac* suppression may not be due to the disabling of particular circuits, but is a general block of seizure-like activity by an overall poorly-transmitting nervous system. It remains surprising that such a putative mechanism of seizure-suppression would be especially effective at reverting *para*^*bss1*^ seizure phenotypes which are severe.

The *cac* gene is one of the most interesting *Drosophila* neurological genes. The gene is predicted to encode 15 annotated transcripts and 14 unique polypeptides. Numerous mutations have been identified (72 alleles) and functions ascribed to different subsets of *cac* mutations [35–37]. Male courtship song alteration is one of the canonical phenotypes of *cac* exemplified by the original *cac*^*S*^ mutation. Subsequently *cac*^*TS2*^ and *cac*^*NT27*^ were also shown to have alterations in courtship song [37]. All of the mutations in this subset show motor defects, seizure-like activity, and behavioral paralysis. These mutations and several other *cac* alleles in this subset all fail to complement each other. The *cac*^*TS2*^ mutation is due to a mis-sense mutation that is thought to alter Ca^++^-dependent inactivation [38]. Thus, although *cac*^*TS2*^ is recessive, it could behave as a gain-of-function mutation. Nevertheless, RNAi experiments presented here show that cac loss-of-function can cause seizure-suppression. However, flies carrying cacRNAi show neither seizure-like activity nor paralysis, suggesting these phenotypes could be due to gain-of-function phenotypes of *cac*^*TS2*^. These issues remain to be determined in future experiments.

Another interesting finding in this study is the co-suppression by *para*^*bss1*^ of the *cac*^*TS2*^ spontaneous seizure-like phenotype induced by high temperature. We presume that this must be due to a loss of spontaneous motoneuron spiking, since activity in the DLM muscle fiber reflects post-synaptic potentials from neuromuscular transmission. The mechanism responsible for this loss of motoneuron spiking is unclear; there are not previously described functions of *para*^*bss1*^ that easily account for it. The *para*^*bss1*^ sodium channel mutation causes gain-of-function phenotypes and leads to hyper-excitability in neurons. It is this hyper-excitability that makes *para*^*bss1*^ mutants more prone to seizures. The *cac*^*TS2*^ mutation causes a less functional Ca^2+^ channel and hence a decrease in release of neurotransmitter. So, bringing two defective ion channels with different effects on membrane excitability effects leads to the suppression of epilepsy. This *Drosophila* suppression resembles seizure-suppression findings in mice [17]. Double mutant mice carrying mutations in two epilepsy genes, Cacna1and Kcna1a showed improvement in both absence epilepsy and limbic seizure phenotypes caused by these mutations [17].

## Materials and Methods

### Fly stocks

*Drosophila* strains were maintained on standard cornmeal-molasses agar medium at room temperature (24°C). The *cacophony (cac)* gene is located on the X chromosome at 10F-11A on the cytological map and encodes a voltage-gated Ca^++^ channel α_1_ subunit implicated in neurotransmitter release [3–8]. The *cac*^*TS2*^ allele is a recessive mutation caused by a substitution (P1385S) at the C-terminus [4]. This position is adjacent to an EF hand motif thought to be involved in calcium dependent inactivation. The *cac*^*TS2*^ mutation causes temperature-sensitive paralysis: apparently due to a reduction, and then loss of synaptic current as the temperature is raised from permissive to restrictive values [4]. The *paralytic (para)* gene is located at map position 1–53.5 and encodes a voltage-gated Na^+^ channel [39–40]. The allele use here is a bang-sensitive (BS) paralytic mutation, *para*^*bss1*^, previously named *bss*^*1*^[14] It is the most seizure-sensitive of fly mutants, the most difficult to suppress by mutation and by drug, and has been proposed as a model for human intractable epilepsy [10]. The *para*^*bss1*^ allele is a gain-of-function mutation caused by the substitution (L1699F) of a highly conserved residue in the third membrane-spanning segment (S3b) of homology domain IV [10]. The *easily shocked (eas)* gene is located at 14B on the cytological map and encodes an ethanolamine kinase [41]. The BS allele used in this study is *eas*^*PC80*^, which is caused by a 2-bp deletion that introduces a frame shift; the resulting truncated protein lacks a kinase domain and abolishes all enzymatic activity [22]. The *slamdance (sda)* gene is located at 97D and encodes an aminopeptidase N. The allele used in this study is *sda*^*is07*.*8*^ caused by a 2-bp insertion in the 5’ untranslated region [42]. The *UAS-cacRNAi* line was obtained from Bloomington *Drosophila* Stock Center. The insert for *UAS-cacRNAi* is located on the 3^rd^ chromosome.

### Double mutants

The double mutants used in this study were constructed by standard genetic crosses and then verified for the presence of both the BS mutation (*sda*, *eas* or *para*^*bss1*^), as well as *cac*^*TS2*^. The presence of *cac*^*TS2*^ in the homozygous double mutant stock was verified by testing for behavioral paralysis after heat shock (37°C for 5 min), which is characteristic for this mutation; BS mutants do not paralyze under such conditions. The presence of the homozygous BS mutation in the double-mutant stocks was verified by backcrossing each double mutant stock to females of the appropriate BS genotype. The progeny from those crosses, which should be homozygous for the BS mutation and heterozygous for *cac*^*TS2*^, were then tested for the BS behavioral phenotype. All of the genotypes arising from the back cross phenotypically resembled BS homozygous flies. The lack of any obvious effects among the different genetic backgrounds tested also indicated the alterations in seizure-sensitivity reported here were due to the homozygous presence of *cac*^*TS2*^ in the double mutant combinations and not likely due to nonspecific genetic background differences.

### BS behavior and heat shock

Behavioral testing for BS paralysis was performed on flies 3d after eclosion, as described previously [16]. Flies were anesthetized with CO_2_ before collection and tested the following day. For testing, 10 flies were placed in a clean food vial and stimulated mechanically with a VWR vortex mixer at maximum speed for 10 s. The *para*^*bss1*^, *eas*, and *sda* mutants ordinarily show 100% penetrance of BS paralytic behavior with this test. Suppression by *cac*^*TS2*^ was initially manifest as a reduced percentage of BS behavioral paralysis in the double mutant compared to the single BS mutant. Recovery from BS paralysis was determined by counting the number of flies standing at different intervals following stimulation. Recovery time was the time at which 50% of flies had recovered from paralysis. For genotypes that display partial penetrance of BS paralysis, only those flies that displayed paralysis were used for recovery time analysis. For BS behavioral analysis, pools of flies are combined for each genotype from among the separate trials (in total, n ≈ 100 for each genotype). For analyses using heat shock (HS), a single fly was placed in a clean food vial and tested the following day. The vial was submerged in a water bath (30°C for 3 min), rested at room temperature (24°C for 30 seconds), and then tested for BS behavioral paralysis. For construction of double mutant stocks, flies were tested similarly for the presence of the *cac*^*TS2*^ mutation except that water bath temperature was 37°C, and the assay was temperature-sensitive behavioral paralysis.

### Electrophysiology

*In vivo* recording of seizure-like activity and seizure threshold determination in adult flies was performed as described previously [16]. Flies 2–3 days post-eclosion were mounted in wax on a glass slide, leaving the dorsal head, thorax, and abdomen exposed. Stimulating, recording, and ground metal electrodes were made of uninsulated tungsten. Seizure-like activity was evoked by high-frequency electrical brain stimulation (0.5 msec pulses at 200 Hz for 300 msec) and monitored by dorsal longitudinal muscle (DLM) recording. During the course of each experiment, the giant fiber (GF) circuit was stimulated by single-pulse electrical brain stimulation and monitored continuously as a proxy for holobrain function. For each genotype tested n ≥ 10.

### Data analysis

Chi-square tests were used to compare the penetrance of seizures. Student’s t-test and ANOVA were used to compare recovery times and seizure thresholds across genotypes, as appropriate. For ANOVA analysis, where the null hypothesis was rejected by the overall F ratio, multiple comparisons of data sets were performed by Fisher’s least significant difference with t-test significance set at P < 0.05. For Figures (1–3 and 5) error bars represents standard error of the mean, and statistical significance is indicated by * P < 0.01, ** P < 0.001 and *** P < 0.0001.

## Supporting Information

S1 Fig Giant fiber stimulation of *cac*^*TS2*^ and bang sensitive mutants. Single pulse electrical stimuli are delivered to the brain (0.2 msec in duration, 0.8 Hz) to activate the giant fiber (GF) neurocircuit. A. GF stimulation comparing DLM responses of *para*^*bss1*^ and *cac*^*TS2*^ single mutants, and a *para*^*bss1*^ *cac*^*TS2*^ double mutant. GF responses are all similar and resemble the wild type GF response. B. GF responses from stimuli delivered at 73 Hz. Upper trace: GF responses in a *sda* mutant occur after each stimulus showing that the GF circuit responds reliably at this frequency. Upper trace: GF responses in a *cac*^*TS2*^ mutant show failures at this stimulation frequency. Arrows show examples of response failures. C. Quantification of synaptic responses shows that sda GF responses show 73 responses without at failure (100% successful GF stimulations at 73 Hz). For *cac*^*TS2*^, only 31 GF responses were elicited by 73 stimulations at 73 Hz (42% successful GF stimulations at 73 Hz). Horizontal calibration: 500 msec for A, 10 msec for B. Vertical calibration: 20 msec for A and B.
(PDF)

S2 Fig Suppression of *eas* bang sensitivity by cacRNAi using different GAL4 drivers. In *eas/Y* hemizygous males without cac-RNAi (white bar), all flies are completely paralyzed by mechanical stimulation. Behavioral paralysis is suppressed using a pan-neuronal GAL4 driver to express cacRNAi (gray bar; genotype: e*lav*^*C155*^*-GAL4 eas/Y;; UAS-cacRNAi/+*). Less effective suppression is observed when cacRNAi is expressed only in GABAergic inhibitory interneurons (black bar; genotype: *eas/Y; GAD-GAL4/+; UAS-cacRNAi/+*) or only in excitatory cholinergic interneurons (striped bar; genotype: *eas/Y; Cha-GAL4/+; UAS-cacRNAi/+*).
(PDF)
