# Correction: Mutations of the Calcium Channel Gene *cacophony* Suppress Seizures in *Drosophila*

**DOI:** 10.1371/journal.pgen.1005871

**Published:** 2016-02-10

**Authors:** The PLOS Genetics Staff

The Data Availability statement for this paper is incorrect. The correct statement is: “All relevant data are within the paper, its Supporting Information files, or available in Dryad via the link: http://dx.doi.org/10.5061/dryad.nc763.” The publisher apologizes for the error.
