# Supplementary figures and images for: Mutations of the Calcium Channel Gene cacophony Suppress Seizures in Drosophila
Source: PLoS Genet. 2016 Jan 15;12(1):e1005784. doi: 10.1371/journal.pgen.1005784 (PMC4714812; doi:10.1371/journal.pgen.1005784)

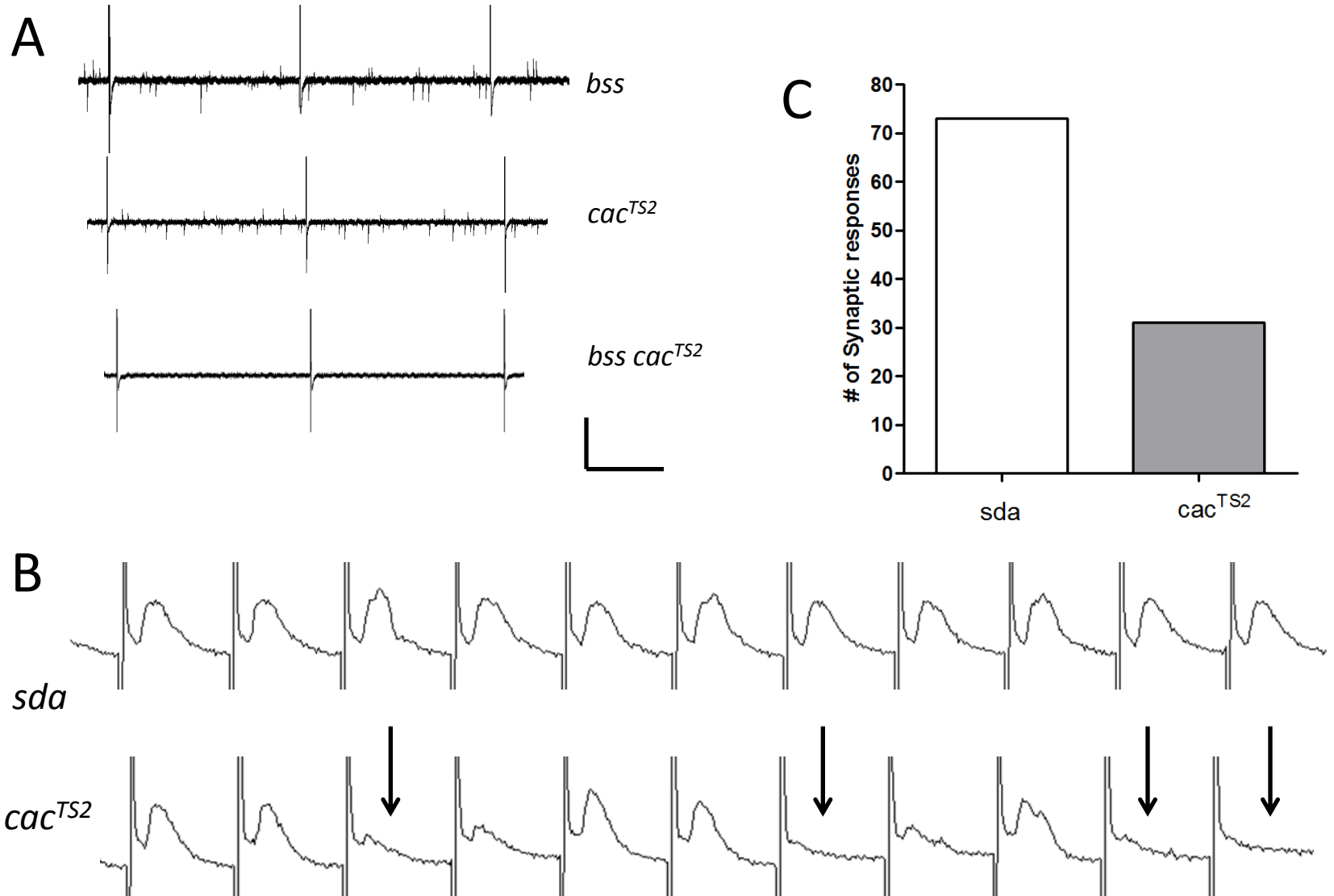

Figure S1.

Supplement: S1 Fig — Single pulse electrical stimuli are delivered to the brain (0.2 msec in duration, 0.8 Hz) to activate the giant fiber (GF) neurocircuit. A. GF stimulation comparing DLM responses of parabss1 and cacTS2 single mutants, and a parabss1 cacTS2 double mutant. GF responses are all similar and resemble the wild type GF response. B. GF responses from stimuli delivered at 73 Hz. Upper trace: GF responses in a sda mutant occur after each stimulus showing that the GF circuit responds reliably at this frequency. Upper trace: GF responses in a cacTS2 mutant show failures at this stimulation frequency. Arrows show examples of response failures. C. Quantification of synaptic responses shows that sda GF responses show 73 responses without at failure (100% successful GF stimulations at 73 Hz). For cacTS2, only 31 GF responses were elicited by 73 stimulations at 73 Hz (42% successful GF stimulations at 73 Hz). Horizontal calibration: 500 msec for A, 10 msec for B. Vertical calibration: 20 msec for A and B. (PDF) [file pgen.1005784.s001.pdf]

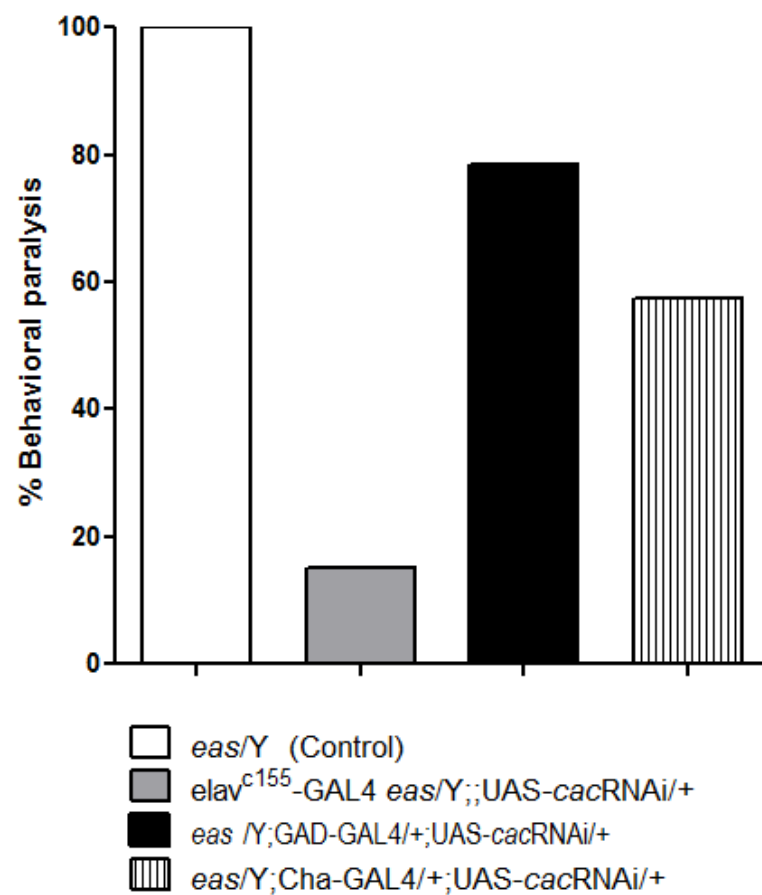

Figure S2.

Supplement: S2 Fig — In eas/Y hemizygous males without cac-RNAi (white bar), all flies are completely paralyzed by mechanical stimulation. Behavioral paralysis is suppressed using a pan-neuronal GAL4 driver to express cacRNAi (gray bar; genotype: elavC155-GAL4 eas/Y;; UAS-cacRNAi/+). Less effective suppression is observed when cacRNAi is expressed only in GABAergic inhibitory interneurons (black bar; genotype: eas/Y; GAD-GAL4/+; UAS-cacRNAi/+) or only in excitatory cholinergic interneurons (striped bar; genotype: eas/Y; Cha-GAL4/+; UAS-cacRNAi/+). (PDF) [file pgen.1005784.s002.pdf]
